# Urgency to treat and early optimized treatment in major depressive disorder: consequences of delayed treatment, barriers to implementation, and practical strategies for clinicians

**DOI:** 10.1017/S1092852925000276

**Published:** 2025-04-14

**Authors:** Oloruntoba J. Oluboka, Jeffrey Habert, Atul Khullar, David J. Robinson, Martin A. Katzman, Larry J. Klassen, Claudio N. Soares, Pratap R. Chokka, Margaret A. Oakander, Roger S. McIntyre, Diane McIntosh, Pierre Blier, Sidney H. Kennedy, Matthieu Boucher

**Affiliations:** 1 Department of Psychiatry, University of Calgary, Calgary, AB, Canada; 2 Department of Family and Community Medicine, University of Toronto, Toronto, ON, Canada; 3 Department of Psychiatry, University of Calgary, Edmonton, AB, Canada; 4 Psychiatry Clinic, Canadian Mental Health Association, London, ON, Canada; 5 START Clinic for Mood and Anxiety Disorders, Toronto, ON, Canada; 6 Adler Graduate Professional School, Toronto, ON, Canada; 7 Northern Ontario School of Medicine, Laurentian and Lakehead University, Thunder Bay, ON, Canada; 8 Department of Psychology, Lakehead University, Thunder Bay, ON, Canada; 9 Eden Mental Health Center, Winkler, MB, Canada; 10 Department of Psychiatry, Queen’s University School of Medicine, Kingston, ON, Canada; 11 Chokka Center for Integrative Health, Edmonton, AB, Canada; 12 Department of Psychiatry, University of Alberta, Edmonton, AB, Canada; 13 Department of Psychiatry and Pharmacology, University of Toronto, Toronto, ON, Canada; 14 Department of Psychiatry, University of British Columbia, Vancouver, BC, Canada; 15 Royal Ottawa Institute of Mental Health Research, University of Ottawa, Ottawa, ON, Canada; 16 Homewood Research Institute, Guelph, ON, Canada; 17 Medical Affairs, Otsuka Canada Pharmaceuticals Inc., Saint-Laurent, QC, Canada; 18 Department of Pharmacology and Therapeutics, School of Biomedical Sciences, Faculty of Medicine and Health Sciences, McGill University, Montréal, QC, Canada

**Keywords:** Adjunctive therapy, major depressive disorder, time-to-treatment, pharmacotherapy, treatment optimization, urgency to treat

## Abstract

Major depressive disorder (MDD) is a serious and often chronic illness that requires early and urgent treatment. Failing to provide effective treatment of MDD can worsen the illness trajectory, negatively impact physical health, and even alter brain structure. Early optimized treatment (EOT) of MDD, with a measurement-based approach to diagnosis, rapid treatment initiation with medication dosage optimization, frequent monitoring, and prompt adjustments in treatment planning when indicated, should proceed with a sense of urgency. In this article, we describe common barriers to providing an EOT approach to treating MDD at each phase of care, along with strategies for navigating these obstacles. Approaching the treatment of MDD with a greater sense of urgency increases the likelihood of symptom reduction in MDD, facilitating full functional recovery and a return to life engagement.

## Introduction

Major depressive disorder (MDD) is a serious and often chronic disease with a lifetime prevalence estimate ranging between 2% and 21% worldwide.^1^ The lifetime prevalence for Canadians is estimated to be 11.3%.^2^ For people with moderate-to-severe MDD, pharmacotherapy is a recommended first-line treatment.^3^ ^–^^5^ Despite this, reports from meta-analyses indicate that only 38%–49% of patients with moderate-to-severe MDD who received either a selective serotonin reuptake inhibitor (SSRI), serotonin-norepinephrine reuptake inhibitor (SNRI), or a tricyclic class of agent for MDD in clinical trials achieved remission in clinical trials.^6^ ^,^^7^ The proportion of patients achieving remission in clinical practice is likely lower.^8^ Findings from a meta-analysis focused on the recognition of MDD by physicians other than psychiatrists suggest that fewer than half of individuals meeting criteria are identified^9^ and, among individuals who received healthcare interventions for a depressive disorder, only 41% received minimally adequate treatment (defined as ≥2 months of pharmacotherapy with ≥4 physician visits or ≥8 psychotherapy sessions within 12 months).^10^ While we recognize the strong support for evidence-based psychotherapies for the treatment of mild-to-moderate MDD,^4^ ^,^^5^ the focus of this review is on optimal pharmacotherapy use.

**Figure 1.**
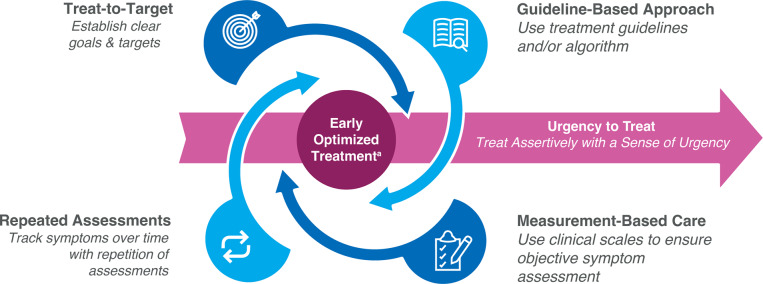
Early optimized treatment and urgency to treat for MDD: how to implement in clinical practice. ^a^Early diagnosis followed by rapid, optimal treatment. MDD, major depressive disorder.

In the management of chronic diseases, such as hypertension or diabetes, rapidly treating to defined therapeutic targets, sometimes using multiple medications, is a common strategy.^11^ ^,^^12^ However, when individuals present with symptoms of an episode of MDD (MDE), the traditional approach of “watchful waiting” may be what clinicians do, along with a “start low and go slow” strategy during treatment management.^13^ ^,^^14^ Used more broadly, these approaches can unfortunately contribute to poor outcomes.^15^ Failure to rapidly address an MDE with a sense of urgency can worsen underlying pathophysiology, increasing symptom severity and reducing the likelihood of treatment success.^16^ ^–^^20^ In contrast to a “slow and sequential” approach to MDD treatment, early optimized treatment (EOT) requires a measurement-based approach to early diagnosis and assessment of symptom severity, rapid treatment initiation and dose optimization, frequent monitoring, and prompt adjustments to a comprehensive treatment plan (Figure 1 ). ^15^ ^,^^16^ With an “urgency-to-treat” (UTT) mindset based on an awareness of the risks of delayed treatment, EOT provides a better opportunity for a full, functional recovery (Supplementary Figure S1). ^15^ ^,^^16^

The use of measurement-based care (MBC), with initial and repeated assessments using validated scales,^21^ provides a proxy for diagnosis of MDD and indicates changes during ongoing management.^22^ Regular follow-up appointments (eg, at 2-week intervals) are critical in monitoring clinical status, optimizing medication dosage, and guiding treatment to symptom remission.^3^ ^,^^5^ Treatment decisions should be implemented using an integrative process, combining clinical experience in conjunction with guideline-based recommendations, such as those in the Canadian Network for Mood and Anxiety Treatments (CANMAT) 2023 Update on Clinical Guidelines for Management of Major Depressive Disorder in Adults.^5^ ^,^^23^ ^–^^25^ Approaching the treatment of an MDE with the mindset of UTT and employing these principles of EOT has been highlighted previously.^15^ ^,^^16^ Nonetheless, many healthcare practitioners (HCPs) are not cognizant of the need for urgent treatment of depressive symptoms or encounter other barriers to implementing an EOT approach to treating people with MDD.^26^ In this review, we highlight the consequences of failing to treat MDD early and urgently, and then consider a range of potential barriers to UTT and EOT in primary care settings and outline how HCPs can navigate these obstacles.

## Consequences of delaying optimal treatment for patients with MDD

Failing to effectively treat an MDE prolongs suffering and can result in demoralization, learned helplessness, and feelings of isolation and loneliness.^27^ Longer durations of MDEs may worsen the trajectory of MDD,^27^ making subsequent episodes more difficult to treat, reducing the chances of achieving remission.^15^ ^,^^20^ ^,^^28^ Individuals enrolled in the Sequenced Treatment Alternatives to Relieve Depression (STAR*D) study who required a greater number of treatment steps (thereby needing more time to achieve remission), had, on average, poorer functional outcomes.^29^ Romera and colleagues found that individuals who were switched to an effective treatment after even 8 weeks of ineffective treatment were less likely to return to their baseline level of functioning compared with those who were switched after 4 weeks.^30^

## End-organ damage

The adage “time is brain” is often applied to the treatment of stroke,^31^ but it is an equally meaningful principle in the treatment of MDD. Untreated MDD is associated with negative effects in multiple brain areas/structures, particularly within the hippocampus^32^ ^,^^33^ (Table 1). Compared with healthy individuals, people with MDD are more likely to have reduced gray matter volume,^32^ ^–^^36^ lower serum levels of nerve growth factor,^37^ and reduced serum brain-derived neurotrophic factor,^38^ a key molecule involved in neurogenesis, neuroplasticity, and synaptogenesis.^39^ ^,^^40^ There is evidence that these effects may increase with duration or chronicity of MDD^35^ ^,^^41^ ^–^^43^ and that they are mitigated by pharmacologic and nonpharmacologic treatment of MDD symptoms^32^ ^,^^44^ ^–^^47^ (Table 1), suggesting that early and rapid treatment may slow or reverse brain changes associated with MDD.

**Table 1.** Associations Between Major Depressive Disorder and Brain Structure, Neurotrophins, and Proinflammatory Markers^32^ ^,^^33^ ^,^^35^ ^–^^38^ ^,^^44^ ^-^^47^ ^,^^58^ ^–^^61^

Parameter	Difference with MDD	Greater difference with DOI/chronicity	Mitigation/reversal with antidepressant treatment^a^
**Gray matter volume**	↓ **Hippocampus** ^32^ ^,^^33^ ^,^^35^	Hippocampus^32^	**Hippocampus** ^32^ ^,^^44^ ^–^^46^
	↓ **Amygdala** ^32^ ^,^^33^	Amygdala^32^	Amygdala^32^ ^,^^45^
	↓ **Frontal cortex** ^33^ ^,^^36^		Frontal cortex^45^
**Neurotrophin levels**	↓ **Peripheral BDNF** ^38^		**Peripheral BDNF** ^47^
	↓ **Peripheral NGF** ^37^		
**Proinflammatory cytokine levels**	↑ **Peripheral TNFα** ^59^		**Peripheral TNFα** ^47^
	↑ **Peripheral IL**-**10** ^59^		**Peripheral IL–10** ^60^
	↑ **Peripheral IL**-**6** ^59^		**Peripheral IL–6** ^60^
	↑ **CSF IL–6** ^58^		
	↑ **Risk of elevated CRP** ^61^		

Note. Meta-analytic results are shown in bold.

BDNF, brain-derived neurotrophic factor; CRP, C-reactive protein; CSF, cerebrospinal fluid; DOI, duration of illness; IL, interleukin; NGF, nerve growth factor; TNFα, tumor necrosis factor alpha.

a Pharmacologic or electroconvulsive therapy.

**Figure 2.**
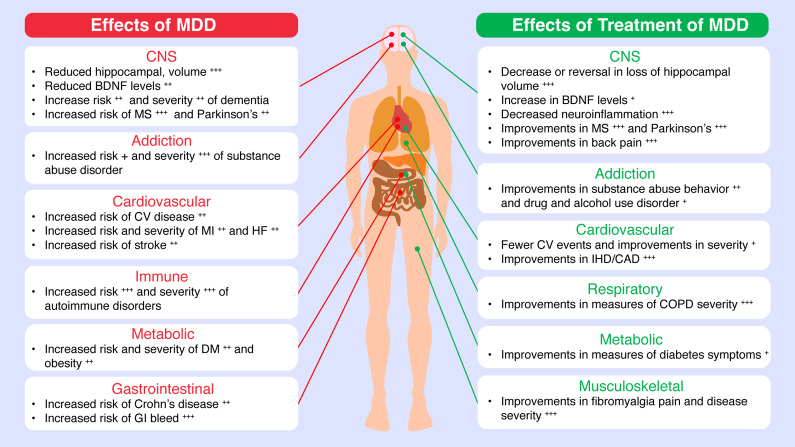
Effects of untreated MDD and of treatment of MDD on brain structure and comorbid conditions. Effects of untreated MDD on somatic conditions are based on Arnaud et al.^50^; effects of the treatment of MDD on somatic conditions are based on Arnaud et al.^51^ Sources for specific effects on brain structure^32^ ^,^^33^ ^,^^35^ ^–^^38^ ^,^^44^ ^–^^47^ ^,^^58^ ^–^^61^ are given in Table 1. BDNF, brain-derived neurotrophic factor; CAD, coronary artery disease; CNS, central nervous system; COPD, chronic obstructive pulmonary disease; CV, cardiovascular; DM, diabetes mellitus; GI, gastrointestinal; HF, heart failure; IHD, ischemic heart disease; MI, myocardial infarction; MS, multiple sclerosis.

Just as poorly controlled diabetes can damage the heart, kidneys, eyes, and other organs,^48^ ^,^^49^ inadequately treated MDD can cause damage throughout the body. In a systematic review, Arnaud et al. presented evidence that MDD is associated with an increased risk of developing a range of cardiovascular, metabolic, autoimmune, and respiratory conditions, as well as worsening preexisting comorbidities^50^ (Figure 2; Supplementary Table S1). Depression is associated with an increased risk of developing or experiencing cardiac arrest, hypertension, myocardial infarction, heart disease, and stroke, and it may worsen the course of preexisting heart failure, myocardial infarction, and stroke.^50^ Depression is also linked to increased risk for or worsening of metabolic conditions (diabetes, obesity, and hyperlipidemia), as well as a range of other conditions including Crohn’s disease, multiple sclerosis, arthritis, asthma, bronchitis, and dementia.^50^ However, just as effective treatment of MDD can mitigate its effects on the brain, effective treatment of MDD also may improve outcomes for comorbidities including heart artery disease and myocardial infarction, multiple sclerosis, Alzheimer disease, Parkinson disease, chronic pain, and chronic lung disease^51^ (Figure 2; Supplementary Table S1).

Inflammation is thought to be an important indirect link between MDD and chronic medical illnesses. For example, findings from numerous studies indicate that an increased risk for coronary heart disease in people with MDD^52^ ^,^^53^ may be mediated, at least in part, by proinflammatory pathways.^54^ ^,^^55^ Circulating inflammatory marker levels are a predictor of coronary heart disease,^56^ ^,^^57^ and inflammatory cytokine levels are significantly elevated in some people with depression compared with healthy controls^47^ ^,^^58^ ^–^^61^ (Table 1). Neuroinflammation is positively associated with severity and duration of depression.^62^ ^,^^63^ Conversely, treatment of MDD is associated with a significant decrease in proinflammatory marker levels^47^ ^,^^60^ (Table 1). MDD treatment also significantly reduces neuroinflammation, with larger decreases observed during longer durations of treatment.^63^ While a direct causal link has not been demonstrated, the positive effects of antidepressant treatment on inflammatory pathways may underlie, or contribute to, improved comorbidity outcomes^51^ in patients with MDD.

## Addressing barriers to UTT and EOT for MDD with practical solutions

Barriers to optimal treatment of MDD can occur at every step of a person’s journey.^26^ Initial barriers include the continuing shortage of primary care providers in Canada and the United States,^64^ ^–^^67^ and individuals’ hesitance to seek treatment even among those with access to services. Barriers to implementation of EOT that HCPs may face in their own practice are summarized in Supplementary Table S2, along with potential strategies for navigating those obstacles. Implementation of the UTT plus EOT approach to treating MDD is illustrated for a hypothetical individual in the Case Scenario (Table 2).

**Table 2.** Possible Case Scenario

Patient	Details			
33-year-old single woman	Medical resident in emergency medicine			
Her father died 2 months earlier; too busy to properly grieve	Places self-worth in her academic and professional successes			
**First episode of MD**				
Triggered by stressful events (job, grief, loneliness)	Affected her mood (pervasive sadness and loss of interest)			
Affected ability to perform in a professional setting	Markedly diminished ability to experience pleasure			
Affected sleep and energy levels				
**Symptoms**				
Depressed mood	Feelings of isolation and loneliness			
Insomnia or hypersomnia	Fatigue or loss of energy			
Feelings of worthlessness or excessive or inappropriate guilt				
**Family history**				
Mother had MDD and anxiety	Brother: ADHD			
**Screening questionnaires**				
MDQ for bipolar disorder^91^	AUDIT for alcohol use disorders^181^			
ASRS for ADHD^182^				
**Diagnosis:** MDD				
**Initial treatment selection:** SNRI (eg, desvenlafaxine 50 mg/d)				
**Measurement** **-based** **assessment**	PHQ–9^a^	SDS	GAD–7	Anxious distress^b^
**Initial scores**	19/27	19/30	14/21	Moderate/severe
**Week 2 follow** **-up** **scores**	12/27	12/30	9/21	Moderate
Percent change from baseline	26%	23%	24%	
**Case evolution**	The patient showed early improvement Aim for continued improvement and response			
**Optimization step**	Increase SNRI to the maximum recommended dose Continue to monitor for effectiveness and tolerability Schedule the next follow-up in 2 weeks to assess			
**Week 4 follow** **-up** **scores**	10/27	10/30	8/21	Moderate
Percent change from baseline	33%	30%	29%	
**Case evolution**	The patient has achieved a partial response with good tolerability Dose is optimized, but she is still far from reaching her treatment goals She is not feeling like her old self and still feels down a lot of times Residual symptoms include irritability and anxiety The patient is open to trying an adjunctive strategy to help her reach her treatment goals			
**Optimization step**	Add on an adjunctive agent (eg, brexpiprazole 0.5 mg/day, to leverage the serotonin1A receptor partial agonism and to address her anxiety) Evaluate her progress and tolerability after 2 to 4 weeks Optimize dose from 1 mg/day to a maximum of 2 mg/day Monitor optimized dose and assess response			

a PHQ.

b Anxious Distress Specifier Interview, DSM-5-TR.

ADHD, attention-deficit/hyperactivity disorder; ASRS, Adult Self-Report Scale for ADHD; AUDIT, Alcohol Use Disorders Identification Test; DSM-5-TR, *Diagnostic and Statistical Manual of Mental Disorders, 5th edition–Text Revision*; GAD-7, Generalized Anxiety Disorder 7-Item; MDQ, Mood Disorder Questionnaire; MDD, major depressive disorder; PHQ-9, 9-Item Patient Health Questionnaire; SDS, Sheehan Disability Scale; SNRI, serotonin norepinephrine reuptake inhibitor.

## Screening and diagnosis

There are numerous reasons why individuals may not seek treatment for depressive symptoms or may avoid reporting their symptoms.^26^ Some people are simply unaware that their symptoms are related to MDD. People often emphasize somatic complaints—lethargy, loss of interest, or difficulty sleeping—rather than depressed mood or anhedonia, making it even more difficult for HCPs to ascribe those symptoms to an MDE.^64^ ^,^^68^ ^–^^71^ While the stigma associated with MDD may be declining,^72^ ^,^^73^ individuals may still experience embarrassment and shame, and be fearful of potential impacts of a diagnosis on their careers.^27^ ^,^^74^ ^,^^75^

Fewer than half of individuals with MDD seen by a physician are recognized as having an MDE.^9^ HCPs often fail to screen for MDD during clinic visits due to time constraints or a perceived lack of resources to treat the condition.^68^ ^,^^76^ ^,^^77^ MDD screening may not even be considered during visits for physical complaints—a consequence of the “one problem, one visit” approach. Other HCPs may not consider MDD screening because they feel they lack the expertise to recognize and treat MDD in its various presentations.^68^ ^,^^76^ ^,^^77^ Among HCPs who do screen for an MDE, a meta-analysis of 41 studies assessing diagnostic accuracy found that only 47% of people were properly detected.^78^ Atypical presentations (eg, somatic complaints) and inability to distinguish symptoms of MDD from other psychiatric conditions may contribute to failure to diagnose and misdiagnosis by HCPs.^79^ ^–^^81^

Clinicians should screen individuals who are at risk for MDD or who present with depressive symptoms.^24^ This does not need to be time consuming or resource intensive, as there are a range of available assessment tools that can be used to rapidly and effectively screen for an MDE.^82^ ^,^^83^ HCPs can start with a simple 2-item questionnaire (Patient Health Questionnaire-2 [PHQ-2]^84^), which screens for the presence of the two cardinal symptoms of an MDE, and helps determine if further assessment and monitoring using the more comprehensive scales is needed.^85^ ^,^^86^ Commonly used scales include the 9-Item Patient Health Questionnaire^21^ (PHQ-9) or the Quick Inventory of Depressive Symptomatology—Self-Report (QIDS-SR).^87^ These and other scales are easy to use and available free online, can be integrated into electronic medical records, and can even be completed prior to an HCP visit.^16^ ^,^^21^ ^,^^82^ ^,^^87^ ^,^^88^ Detailed summaries of screening/monitoring tools can be found in Oluboka et al.^16^ and in the CANMAT 2023 Update.^5^

## Alternative diagnoses and comorbidities

The diagnosis of MDD also requires that other conditions that present with depressive symptoms are considered (Supplementary Table S3).^24^ ^,^^79^ ^,^^89^ A substantial proportion of people who initially receive a diagnosis of MDD later receive a different or additional diagnosis.^79^ For individuals who have depressive symptoms that are not responding to the current treatment plan, HCPs should continue to explore alternative diagnoses in subsequent visits. The criteria for an MDE occurring in bipolar disorder (bipolar depression) are identical to those required to diagnose an episode in MDD.^90^ Therefore, it is crucial to consider this as a potential diagnosis, and screen for the presence of bipolar disorder. This can be done with the Mood Disorder Questionnaire (MDQ),^91^ Rapid Mood Screener,^92^ or Bipolarity Index.^93^

Untreated and unrecognized psychiatric comorbidities can affect the treatment and prognosis of MDD and, therefore, identifying these conditions at the earliest possible point is critical for treatment planning. It has been estimated that 46% of people with MDD have a comorbid anxiety disorder,^94^ and anxious distress occurs in up to 78% of people with MDD.^95^ The co-occurrence of an anxiety disorder during an MDE is associated with significantly reduced quality of life, increased suicidal ideation, greater healthcare resource utilization, and greater medical expenditures compared with an MDE without comorbid anxiety.^18^ ^,^^94^ ^,^^96^ ^,^^97^ Individuals with more severe baseline anxiety may be less responsive to MDD treatment.^98^ When diagnosed, comorbid anxiety and anxious distress may be treated effectively with first-line medications indicated for MDD,^5^ ^,^^14^ ^,^^99^ potentially with the use of adjunctive therapy.^100^ Attention-deficit/hyperactivity disorder (ADHD) is also a common comorbidity,^101^ occurring in an estimated 7% (95% CI, 4%–11%) of adults with MDD.^102^ MDD with comorbid ADHD is associated with more severe and chronic depressive symptoms, poorer functional outcomes, and an increased risk of suicide compared with MDD alone.^101^ ^,^^103^ ^–^^106^ Individuals who receive active ADHD treatment, however, are no more likely to be resistant to MDD treatment than those without ADHD.^103^ Psychiatric comorbidities commonly encountered in people with MDD for which CANMAT has provided treatment recommendations include anxiety disorders, substance use disorders, ADHD, and personality disorders.^107^

Nonpsychiatric comorbidities also can complicate the course and treatment of MDD. As an example, sleep fragmentation associated with obstructive sleep apnea, which occurs in more than 30% of individuals diagnosed with MDD,^108^ can lead to daytime sleepiness, fatigue, and impaired memory and cognition.^109^ People with MDD have longer obstructive apneic episodes compared with nondepressed individuals, resulting in substantially greater hypoxia^110^ and neuronal damage.^111^ However, detecting comorbid obstructive sleep apnea and treating it with continuous positive airway pressure therapy can significantly reduce symptoms of depression in people with MDD.^112^ CANMAT treatment recommendations for other common metabolic and medical comorbidities in people with MDD are available.^107^

## Increasing awareness and reducing stigma

Public awareness and mental health literacy are keys to reducing stigma associated with MDD and hesitancy to seek treatment.^27^ ^,^^113^ ^,^^114^ Internet-based programs have the potential to increase mental health literacy and may encourage users of online materials to seek appropriate treatment.^113^ An assessment of web-based interventions revealed that those based on structured programs that target specific populations and include evidence-based information can improve mental health literacy, reduce stigma, and increase healthcare seeking.^114^ Exposure to such interventions may open individuals to recognizing their own symptoms and discussing them with their HCPs. Clinicians can also help increase understanding and reduce stigma associated with an MDD diagnosis by directing people to a peer support group (eg, Hope+Me, Mood Disorders Association of Ontario, Peer Support Canada) or interactive web resources (eg, MoodFX^115^), and providing patient education regarding the nature of MDD and its treatment.^27^ The CANMAT Health Options for Integrated Care and Empowerment in Depression (CHOICE-D) Patient and Family Guide to Depression Treatment,^116^ a plain-language guide to depression treatment based on the CANMAT clinical treatment guidelines^3^ ^,^^24^ ^,^^117^ ^–^^121^ and written by patients and families, is another important tool for improving understanding of MDD treatment options and encouraging greater patient involvement in healthcare decision-making.^116^ ^,^^122^ The CANMAT Depression Guidelines^5^ provide a comprehensive guide to managing persons with MDD at all stages.

## Employing UTT + EOT for MDD

### Prescribing medication

The traditional “watchful waiting” approach is a critical barrier to EOT.^15^ When moderate-to-severe MDD is diagnosed, the EOT approach involves the rapid initiation of an antidepressant medication that carries a first-line recommendation (which is generally a monoamine reuptake inhibitor).^3^ ^,^^5^ A combination of pharmacotherapy and evidence-based psychotherapy may be considered.^5^ The initial medication choice ideally considers factors that impact long-term tolerability, such as the likelihood of sexual dysfunction, sedation, and potential for weight gain.^123^ The individual’s symptoms and comorbidities also impact medication choices that ideally increase the likelihood of early response.^5^ For example, a person struggling with MDD with anxious distress and/or a comorbid anxiety disorder could benefit from a prescription that has a higher likelihood of addressing these symptoms.^3^ ^,^^14^ HCPs should consider any history of response or nonresponse to previous medications^3^ and discuss options with their patients, addressing the acceptability, or lack thereof, of potential adverse effects.

### Early follow-up and medication optimization

Older, now outdated guidelines supported extended medication trials in MDD, advocating up to 8 weeks of antidepressant treatment before any dose optimization, medication augmentation, or switch options were considered.^124^ ^–^^126^ However, most people will require an earlier adjustment to their medication,^79^ and overly long trials of a particular agent without timely follow-up is inconsistent with optimal MDD treatment. HCPs may hesitate to optimize medication doses rapidly, instead making incremental changes after each failed trial.^15^ ^,^^16^ This “go-slow” approach may increase the time to remission and reduces the likelihood of full, functional recovery.^15^ Consequently, a much earlier reassessment of symptoms (eg, 1–4 weeks after initiation of pharmacotherapy^3^ ^,^^5^) using MBC^3^ ^,^^5^ is a critical part of UTT + EOT (Figure 1).

Applying MBC ensures a systematic approach to symptom assessment at baseline and in measuring response to treatment. Although clinicians may have concerns about adequate time and resources for monitoring symptoms, MBC actually helps streamline/organize care to make it more efficient and effective. Together with MDD symptom assessment scales (eg, the PHQ-9 or QIDS-SR), other subjective scales are available that assess the degree of functional impairment (eg, the Lam Employment Absence and Productivity Scale,^127^ Sheehan Disability Scale [SDS]^128^ or the World Health Organization Disability Assessment Scale, self-rated^129^), which helps set treatment goals beyond symptom remission.^25^

In clinical trials, a lack of improvement in symptoms or functioning in the first 2 weeks after treatment initiation is predictive of later treatment failure.^15^ In line with these findings, clinical guidelines now recommend reassessing individuals within 2 weeks of starting medication to look for early symptom reduction and improved functional capabilities, to monitor adverse effects, and to address dosage optimization.^3^ ^,^^5^ Repeated follow-up every 2 to 4 weeks thereafter is recommended to assess the need for further refining of the treatment plan, based on the HCP’s clinical judgment (with the assistance of assessment scores).

**Figure 3.**
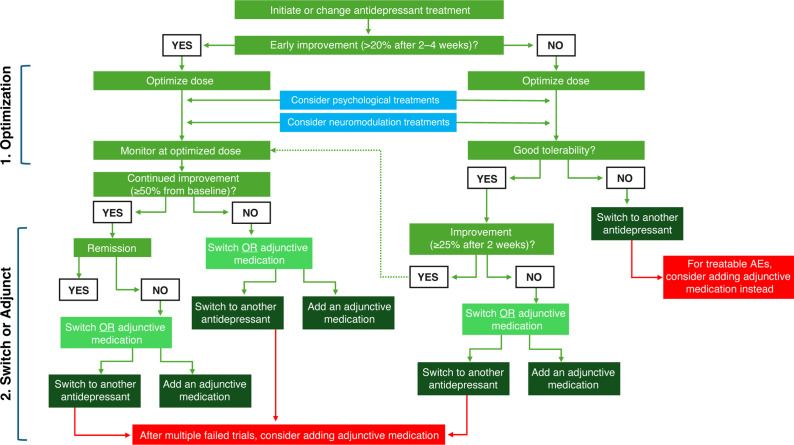
An algorithm for guiding treatment steps in the management of MDD, based in part on the 2023 update on CANMAT clinical guidelines. Adapted with permission from Lam et al.^5^ CANMAT, Canadian Network for Mood and Anxiety Treatments; PHQ-9, 9-Item Patient Health Questionnaire.

For HCPs who find the process of rapid treatment optimization daunting, clinical practice guidelines and algorithms are valuable resources to guide subsequent treatment adjustment decisions during follow-up appointments.^3^ ^,^^5^ ^,^^24^ ^,^^130^ An algorithm developed by the authors (Figure 3) and generally based on the updated CANMAT guidelines may support HCP decision-making for treatment optimization. The first step at the (EOT recommended) 2-week follow-up is to increase medication dosage until either no further clinical benefit has been realized, or side effects have become unacceptable.^3^ If insufficient improvement has occurred, (ie, <25% reduction in scale scores), an add-on/adjunctive medication may be needed^131^ (Supplementary Table S4). Alternatively, a medication switch can be considered, particularly when tolerability is a problem, although in some cases, the addition of an adjunctive medication can minimize or reverse adverse effects.^5^ Strategies for addressing “difficult-to-treat depression”^5^ ^,^^132^ ^,^^133^ are discussed below.

### Therapeutic alliance and adherence

HCPs may find that a lack of therapeutic alliance hinders their ability to engage people in the development of a treatment plan and ensure their adherence once developed.^134^ The maxim, “drugs don’t work in people who don’t take them,” credited to former US Surgeon General C. Everett Koop,^135^ aptly sums up the consequences of nonadherence, which can have many underlying causes. Some individuals may simply lack insight into their symptoms and/or feel that they do not need medication. Others may fail to fill prescriptions if they cannot afford them. Patients may have concerns about adverse effects that were not adequately addressed or may overestimate the risks or severity of such adverse effects. Still others may believe that there is a stigma regarding taking a medication for mental health conditions.^64^ ^,^^136^ ^–^^138^

Fewer than half of individuals prescribed a medication for MDD remain adherent (defined as taking the medication on ≥80% of days covered) after 3 months, and only a quarter are adherent at 1 year.^139^ Sexual dysfunction and weight gain are among the most common reasons for stopping antidepressant medication because these adverse effects are described as “extremely difficult to live with,”^123^ although people are often hesitant to discuss these issues. Some individuals stop their medication due to a perceived lack of improvement, while others stop once their symptoms improve, believing that they no longer need the medication.^64^ ^,^^134^ ^,^^137^ Lack of trust in the HCP and/or poor patient–HCP communication can also contribute to poor adherence to the treatment plan.^64^

While MBC is central to the UTT + EOT approach, successful treatment also hinges on an open and supportive relationship with the patient.^21^ ^,^^64^ When people with an MDE were asked what they valued in their care, the most highly ranked items included having trust in the HCP’s goals for their care and having a feeling that their HCP was listening to and supporting their own goals and concerns.^140^ People also regarded receiving information about their treatment and knowing what to expect as important aspects of their depression care.^140^

To ensure individuals’ confidence in the treatment plan and support their adherence to treatment, HCPs can spend a few minutes at the time of the initial prescription and at subsequent treatment steps educating their patients. They should let them know what to expect from the medication, including any potential early (and usually transient) side effects, and emphasize the importance of adherence.^141^ ^,^^142^ HCPs should routinely inquire about adherence and tolerability, proactively discussing issues such as sexual dysfunction, risk of sedation, and weight gain.^123^ They should encourage patients to continue taking medication regularly to increase the likelihood that symptoms will continue to improve over time.

### Providing treatment that persists to symptomatic remission and full functional recovery

When a person with type 2 diabetes or hypertension initiates treatment, HCPs will continue to optimize their regimen to reach defined treatment targets^11^ ^,^^12^ Analogously, treatment goals for MDD should aim beyond improvement of the presenting symptoms to include a full symptomatic remission and return to full functioning.^23^ ^,^^143^ ^–^^145^ Some patients may become nonadherent to MDD treatment after symptoms have partially improved. Likewise, HCPs may stop optimizing treatment after a partial response.

Many of the same barriers that hinder the UTT + EOT approach in the treatment of acute MDD also impede the continuation of treatment to full functional recovery. Ongoing symptoms of unaddressed psychiatric or somatic comorbidities, lack of social or psychological support outside of the clinic (which may include treatment stigmatization), and challenges with returning to work or lack of employment can limit a person’s commitment to continued treatment.^64^ ^,^^134^ Costs and limited coverage for both pharmacologic and psychological treatments can also be barriers to continuing MDD treatment.^64^ For HCPs, barriers to continued treatment to full functional recovery may include limited awareness of available counseling resources.

### Continuing measurement-based care

Persistence in treating to full functional recovery requires the HCP to continue treatment and monitor progress using MBC until that target is achieved.^15^ ^,^^16^ For an MDE, the treatment target is a PHQ-9 score target of <5 for symptom remission^21^ and an SDS total score of ≤6 for functional recovery.^146^ If an individual has more than 50% improvement from treatment initiation, but still has a PHQ-9 score of 6 to 8, the HCP should continue to target the remaining symptoms and functional deficits that remain.^146^ Patient-reported outcome scales (eg, the 12-Item Short-Form Health Survey^147^) may also be used to assess specific aspects of well-being that the individual feels are important measures of their own treatment success.^144^ ^,^^145^ ^,^^148^ Because functional recovery can lag behind symptomatic remission,^149^ ^–^^151^ the clinician should continue to monitor function and subjective outcomes after the symptom reduction target is met.^23^ ^,^^24^ ^,^^152^ Continuing measurement-based treatment of comorbid conditions is also critical for achieving full functional recovery.

Even with the vigorous application of the UTT + EOT approach, some people will have suboptimal outcomes and will be unable to achieve sustained remission, despite multiple treatment steps.^132^ ^,^^133^ In these individuals with “difficult-to-treat depression,” treatment goals may be revised to controlling symptoms and maximizing function, as in the management of other chronic diseases (Supplementary Table S5).^5^ ^,^^132^ ^,^^133^

### Medication combinations

Some HCPs may be hesitant to combine medications in the treatment of an MDE, but for individuals with an inadequate response to antidepressant treatment and for those with psychotic symptoms, psychomotor agitation, or hostility, medication combinations may be necessary.^153^ ^,^^154^ The use of combination pharmacotherapies^131^ ^,^^155^ ^,^^156^ including augmentation with low-dose adjunctive medications initially indicated for psychosis (ie, serotonin-dopamine modulators [SDMs]),^157^ ^,^^158^ have shown efficacy for treating people with MDD,^5^ with or without psychotic features.^5^ ^,^^159^ Aripiprazole and brexpiprazole are first-line adjunctive agents recommended in the CANMAT 2023 Update^5^ (Supplementary Table S4). Clinicians should consider these options early in treatment, using medications with different receptor affinities to target specific symptoms.^3^ ^,^^153^ ^,^^160^ ^–^^162^ Adjunctive treatment with an SDM is associated with significant reductions in healthcare resource utilization,^163^ and earlier initiation of the SDM after beginning antidepressant treatment (mean of 3.9 months^163^ or 0–6 months^164^) results in greater reductions in healthcare costs and resource utilization compared with later initiation. Adjunctive agents that also have approved indications for psychotic disorders are generally prescribed at lower doses for the treatment of mood disorders. When considering the addition of an adjunctive agent, clinicians must use caution and discuss with their patients the potential benefits versus adverse effects of these options, which also includes addressing the potential stigma that these medications might carry.^138^ ^,^^165^

### Nonpharmacologic strategies

This discussion of strategies for the UTT + EOT approach has focused on pharmacologic treatment of MDD. However, many people will not attain remission and a functional recovery with pharmacologic treatment alone. Nonpharmacologic strategies^5^ are an essential component of treatment planning, and should be implemented with the initiation of pharmacotherapy. MDD drastically affects all areas of a person’s life, and patients should be encouraged to take an active part in their recovery while pharmacotherapy is being optimized. For example, gentle behavioral activation gained from activities such as attending to personal hygiene, taking short walks, or brief periods of socialization can provide a sense of autonomy and control, as opposed to solely taking medication and passively waiting for it to work.

Evidence-based guidelines recommend the use of nonpharmacologic strategies for mild-to-moderate MDD and combined pharmacologic and nonpharmacologic strategies for other individuals, particularly those with moderate-to-severe MDD.^24^ ^,^^162^ Cognitive behavioral therapy (CBT), interpersonal therapy (IPT), and behavioral activation (BA) are first-line recommended psychological therapies for the acute treatment of MDD. CBT and mindfulness-based cognitive therapy are first-line recommendations for maintenance treatment.^120^ In addition to psychotherapies, repetitive transcranial magnetic stimulation (rTMS) has shown efficacy when combined with antidepressant pharmacotherapy.^5^ ^,^^166^

Emerging technologies can play an important role in reducing the burden on time and resources for patients and HCPs in MDD care.^167^ The CANMAT Guidelines^5^ provide an overview of guided and unguided apps to provide CBT and other therapies as adjuncts to standard care. A 39-study meta-analysis indicated that internet-based programs can personalize CBT and reduce depressive symptoms (based on PHQ-9 score) versus merely being placed on a wait list.^168^ Other applications (eg, Text4Mood) can provide supportive CBT-based messaging to people with MDD,^169^ or may be valuable in addressing specific symptoms or comorbidities. As an example, MoodGYM has been shown to provide substantial improvement in anxious symptoms in people with MDD.^170^ Cognitive behavioral therapy for insomnia (CBTi), which reduces Insomnia Severity Index scores compared with hypnotics or no treatment in people with an MDE,^171^ also can be delivered effectively via the Internet.^172^ The 2016 CANMAT guidelines section on disease burden and principles of care provides additional examples of internet-based resources.^24^ HCPs are cautioned that online applications may not be evidence-based or may fail to ensure data security or patient privacy and safety.^173^ ^,^^174^ When recommending an online program, HCPs should ask at subsequent visits if the program was adopted and whether the patient found it useful.

Additional recommended nonpharmacologic approaches that can be used in conjunction with pharmacotherapy, include lifestyle interventions such as exercise and light therapy.^5^ ^,^^121^ Exercise is clearly associated with substantial symptom reduction in people diagnosed with MDD.^175^ It is recommended as first-line therapy for mild-to-moderate MDD and as a second-line adjunctive treatment option for moderate-to-severe MDD.^121^ In meta-analyses, exercise effectively reduced symptoms of depression in people with MDD compared with physically inactive controls^176^ and, when used adjunctively, was significantly more effective than pharmacotherapy alone.^177^ Evidence suggests that just 90 minutes a week of physical activity is enough to have a positive impact on mood symptoms and cognition in individuals with serious mental illness, and time exercising was significantly associated with brain-derived neurotrophic factor expression in study participants.^178^ Light therapy was associated with statistically significant mild-to-moderate reduction in symptoms of nonseasonal depression in a meta-analysis of 23 randomized controlled trials.^179^ It is recommended as a second-line lifestyle intervention for mild severity nonseasonal MDE.^5^ Finally, clinicians can provide information to support their patients with MDD within the community, including resources for accessing social support agencies (eg, for food, housing, or domestic violence issues) and faith communities, where appropriate. Even among people who are not socially isolated, loneliness can have long-lasting, negative effects on depressive symptom severity and the risk of a subsequent MDE.^180^ Therefore, providing support for reducing social isolation can be an important component of care for people with MDD.

## Conclusions

The consequences of delaying effective treatment for an MDE are grave. People with MDD and their HCPs encounter numerous obstacles to the urgent and optimal treatment of MDD. A lack of awareness of an UTT + EOT approach is the first of these obstacles, and we hope that this article addresses that educational need. Suboptimal treatment of MDD can worsen illness trajectory, as well as negatively impact physical health and alter brain structure and function. HCPs who implement strategies to overcome barriers to the UTT + EOT approach of using early diagnosis, rapid treatment initiation, frequent measurement-based monitoring, prompt adjustment of treatment, and timely medication combination, may improve patient outcomes.

## Supporting information

Oluboka et al. supplementary material Oluboka et al. supplementary material

## Data Availability

Data contained in this review article are available from the cited sources.

## Supplementary material

The supplementary material for this article can be found at http://doi.org/10.1017/S1092852925000276.
