# Supplementary material for: Urgency to treat and early optimized treatment in major depressive disorder: consequences of delayed treatment, barriers to implementation, and practical strategies for clinicians
Source: CNS Spectr. 2025 Apr 14;30(1):e54. doi: 10.1017/S1092852925000276 (PMC13064778; doi:10.1017/S1092852925000276)
Supplement: Oluboka et al. supplementary material [file S1092852925000276sup001.pdf]

SUPPLEMENTAL MATERIALS

URGENCY TO TREAT AND EARLY OPTIMIZED TREATMENT IN MAJOR DEPRESSIVE DISORDER: CONSEQUENCES OF DELAYED TREATMENT, BARRIERS TO IMPLEMENTATION, AND PRACTICAL STRATEGIES FOR CLINICIANS

Oloruntoba J. Oluboka, MD[1]*; Jeffrey Habert, MD, CCFP, FCFP[2]*; Atul Khullar, MD, MSc, FRCPC, DABPN, FAASM[3]; David J. Robinson, MD, FRCPC[4]; Martin Katzman, MD, FRCPC[5,6,7,8]; Larry J. Klassen, MD, FRCPC[9]; Claudio N. Soares, MD, PhD, MBA, FRCPC[10]; Pratap R. Chokka, MD, FRCPC[11]; Margaret A. Oakander, MD FRCPC[1]; Roger S. McIntyre, MD, FRCPC[12]; Diane McIntosh, MD, FRCPC[13]; Pierre Blier, MD, PhD[14]; Sidney H. Kennedy, MD, FRCPC[15], Matthieu Boucher, PhD[16-17]

[1]Department of Psychiatry, University of Calgary, Calgary, AB, Canada; [2]Department of Family and Community Medicine, University of Toronto, Toronto, ON, Canada; [3]Department of Psychiatry, University of Calgary, Edmonton, AB, Canada; [4]Psychiatry Clinic, Canadian Mental Health Association, London, ON, Canada; [5]START Clinic for Mood and Anxiety Disorders, Toronto, ON, Canada; [6]Adler Graduate Professional School, Toronto, ON, Canada; [7]Northern Ontario School of Medicine, Laurentian and Lakehead University, Thunder Bay, ON, Canada; [8]Department of Psychology, Lakehead University, Thunder Bay, ON, Canada; [9]Eden Mental Health Center, Winkler, MB, Canada; [10]Department of Psychiatry, Queen’s University School of Medicine, Kingston, ON, Canada; [11]Chokka Center for Integrative Health, Edmonton, AB, Canada, and University of Alberta, Edmonton, AB, Canada; [12]Department of Psychiatry and Pharmacology, University of Toronto, Toronto, ON, Canada; [13]Department of Psychiatry, University of British Columbia, Vancouver, BC, Canada; [14]Royal Ottawa Institute of Mental Health Research, University of Ottawa, Ottawa, ON, Canada; [15]Homewood Research Institute, Guelph, ON, Canada; [16]Medical Affairs, Otsuka Canada Pharmaceuticals Inc., Saint-Laurent, QC, Canada; [17]Department of Pharmacology and Therapeutics, School of Biomedical Sciences, Faculty of Medicine and Health Sciences, McGill University, Montréal, QC, Canada

Supplemental Table 1. Effects of Untreated MDD and of Treatment of MDD on Comorbid Conditions.

Adapted with permission from Arnaud et al. 2022[1] and Arnaud et al. 2023[2]

Body system	Comorbidity	Increased risk of comorbidity	Increased severity of comorbidity	Improvement with MDD treatment
**Nervous**	Dementia and/or Alzheimer's disease	✓✓	✓✓	✓
	Parkinson's disease	✓✓	—	✓✓✓
	MS	✓✓✓	✓	✓✓✓
	Headache	✓✓	X	✓
	Chronic pain	—	X	✓
	Epilepsy	X	—	
**Cardiovascular**	Cardiovascular disease	✓✓	✓	✓
	Ischemic heart disease/CAD	✓✓	✓	✓✓✓
	MI	✓✓	✓✓	✓
	Heart failure	✓✓	✓✓✓	—
	Hypertension	✓✓	X	X
	Stroke	✓✓	✓	X
**Gastrointestinal**	GI hemorrhage	✓✓✓	X	—
**Metabolic**	Diabetes mellitus	✓✓	✓✓	✓
	Metabolic syndrome	✓	✓	—
	Obesity	✓✓	✓✓	—
	Hyperlipidemia	✓✓	✓	—

**Autoimmune**	Autoimmune diseases	✓✓✓	✓✓✓	—
	HIV/AIDS	✓✓	✓✓	—
	SLE	✓✓✓	—	—
	Psoriasis	✓✓✓	X	—
	Crohn's disease	✓✓	✓	—
	Ulcerative colitis	X	X	—
**Skeletal**	Osteoporosis	✓✓✓	—	—
	Arthritis	✓✓	✓	—
	Joint disorder	X	—	—
	Fibromyalgia	—	—	✓✓✓
**Respiratory**	Asthma	✓✓	✓✓	—
	Bronchitis	✓✓✓	✓✓✓	—
	COPD	—	—	✓✓✓
**Addiction disorders**	Substance abuse disorder	✓	✓✓✓	✓✓
	Drug use disorder	✓✓	✓✓✓	✓
	Alcohol use disorder	✓	✓✓	✓
**Cancer**		✓	X	X

✓ = mix of significant and non-significant associations.
✓✓= associations in at least some subgroups found in most studies.
✓✓✓= associations found in all studies.
X = no associations found in most studies.
— = not studied.
CAD, coronary artery disease; COPD, chronic obstructive pulmonary disease; GI, gastrointestinal; HIV/AIDS, human immunodeficiency virus or acquired immune deficiency syndrome; MDD, major depressive disorder; MI, myocardial infarction; MS, multiple sclerosis; SLE, systemic lupus erythematosus.

Supplemental Table 2. Barriers to Successful Early Optimized Treatment and Solutions for Clinicians

Barrier	Solution(s)
**Screening and diagnosis**	
Lack of awareness of need	• Public awareness/mental health literacy programs<br>• Physician education
Lack of access to HCP/primary care team	• Regional entities (eg, CPSA and Alberta Medical Association) provide people access to HCPs who are accepting patients in their region<br>• Systemic solutions (eg, increase number of family medicine practitioners via increases in pay/equitable pay structure similar to that in other specialties; subsidies for increased costs) are needed
Missing early diagnosis	• Screening for patients at risk<br>• Awareness of atypical presentations (eg, somatic complaints)
Discomfort with/lack of resources for diagnosing MDD and comorbidities	• Use digital screening tools, such as EarlyDetect screen for MDD and some of its common comorbidities
Misdiagnosis (of MDD in people with bipolar II disorder)	• Continue to reevaluate alternative diagnoses (eg, bipolarity/substance use disorder)<br>• If suspect, screen with MDQ, RMS, ASRS
Failure to identify and treat comorbid condition(s)	• Be aware of and on the lookout for common comorbidities<br>• Be open-minded to possibilities (especially anxiety, ADHD, substance abuse)<br>• Use digital screening tools such as EarlyDetect<br>• Conduct basic laboratory work followed by referral if indicated
Stigma of the diagnosis	• Peer support groups (eg, Peer Support Canada, Hope+Me in Ontario)<br>• Patient education (eg, CHOICE-D)
**Employing UTT + EOT for MDD**	

"Wait-and-see" and "go-slow" approaches	• Understand the effects associated with duration of untreated MDD<br>• Treat with urgency to mitigate effects on trajectory of illness and overall health
Suboptimal dosing	• Monitor for improvement over first weeks of treatment using measurement-based care<br>• Optimize dose without hesitation<br>• Consider increasing dose in absence of unacceptable adverse effects
Suboptimal medication	• Use add-on medications as needed based on measurement-based care<br>• Be ready to augment or switch<br>• Use pharmacologic and psychological or interventional treatment modalities (eg, ketamine, ECT, deep brain stimulation, rTMS)
Discomfort with combining medications	• Education on value of adjunctive medications, such as SDMs (formerly "atypical antipsychotics"), in combination approaches earlier in treatment<br>• Prioritize add-on medications over switching unless there are patient preference or tolerability issues
Time constraints	• Use algorithms to save time, create efficiency<br>• Consider flexible hours, remote appointments, self-monitoring
Lack of resources for follow-up	• Follow-up is critical for proper treatment; requires better integrated care
Lack of therapeutic alliance	• Listen more intently to individual's concerns<br>• Provide patient education (CHOICE-D)
Nonadherence to treatment	• Patient education on the benefits of treatment, insisting on the consequence of avoiding treatment and/or non-adherence
Filling first prescription	• Check individuals' access to drug plan and ability to pay for prescriptions<br>• Maintain knowledge of patient support programs; if people cannot afford the medication, they will not fill the prescription<br>• Provide early education, anticipating potential areas of concern

	• Anticipate messaging from pharmacist on “antipsychotic” medicines without proper information about their established role at lower doses for MDD
Continuing medication	• Choose a drug with a tolerability profile acceptable to the individual<br>• Inform patients of potential tolerability issues at initiation
**Providing treatment that persists to symptomatic remission and full functional recovery**	
Lack of persistence to full symptomatic and functional recovery	• Do not stop optimizing until the individual achieves full functional recovery<br>• Continue using measurement-based care: use PHQ-9 to assess symptoms and SDS to assess function at treatment initiation and follow-up visits<br>o Targets: PHQ-9, <5; SDS total score, ≤6
Lack of knowledge about counselling resources in community	• Keep a list of patient resources readily available. Examples:<br>o Canadian Mental Health Association (in person or online courses)<br>o Anxietycanada.com<br>o MoodGYM<br>o SAMHSA national helpline<br>o Recommended books (eg, *Feeling Good* by David Burns; *This Is Depression: A Comprehensive, Compassionate Guide for Anyone Who Wants to Understand Depression* by Diane McIntosh)<br>o Relevant podcasts/social media
Difficult-to-treat depression[3]	• Achieve optimal symptom control using measurement-based treatment<br>• Target symptoms associated with poor outcome<br>• Target symptoms to maximize function and quality of life<br>• Manage comorbidities to reduce overall symptom burden<br>• Optimize long-term outcome by ensuring adequate prophylaxis<br>• Use self-management techniques to empower patients

	• Use integrated mental health services to help provide a sense of containment and ensure wide consideration of treatment options<br>• Establish regular review of the individual’s diagnosis and treatment

ADHD, attention-deficit/hyperactivity disorder; ASRS, Adult Self-Report Scale; CHOICE-D, Canadian Network for Mood and Anxiety Treatments Health Options for Integrated Care and Empowerment in Depression; CPSA, College of Physicians & Surgeons of Alberta; ECT, electroconvulsive therapy; EOT, early optimized treatment; HCP, healthcare practitioner; MDD, major depressive disorder; MDQ, Mood Disorder Questionnaire; PHQ-9, 9-Item Patient Health Questionnaire; RMS, Rapid Mood Screener; rTMS, repetitive transcranial magnetic stimulation; SAMHSA, Substance Abuse and Mental Health Services Administration; SDM, serotonin-dopamine modulator; SDS, Sheehan Disability Scale; UTT, urgency to treat.

Supplemental Table 3. Common Psychiatric Conditions That Can Present With Symptoms Shared With Those of Major Depressive Disorder (Based on DSM-5 Differential Diagnosis)

**Shared or Similar Symptoms**	**Condition(s)**
Mood disturbance	Mood disorder due to another medical condition; substance/medication-induced depressive or bipolar disorder (in the context of withdrawal)
Depressed mood; persistent negative emotional state; persistent inability to experience positive emotions	Persistent depressive disorder (dysthymia); premenstrual dysphoric disorder; body dysmorphic disorder; dissociative identity disorder; neurocognitive disorders; posttraumatic stress disorder
Decreased interest in usual activities; diminished interest in significant activities	Premenstrual dysphoric disorder; posttraumatic stress disorder
Lack of sexual interest/arousal	Female sexual interest/arousal disorder; male hypoactive sexual desire disorder
Anxiety	Anxiety disorders; premenstrual dysphoric disorder; cannabis use disorder
Significant impairment in social, occupational, or other important areas of functioning; concern about being negatively evaluated by others	Adjustment disorder; social anxiety disorder
Reluctance to leave home	Anxiety disorders; agoraphobia
Rumination	Obsessive-compulsive disorder; illness anxiety disorder
Irritability; irritable mood; low frustration tolerance	ADHD; bipolar I disorder; bipolar II disorder; premenstrual dysphoric disorder; manic episodes with irritable mood or mixed episodes

Inability to concentrate; problems with concentration; distractibility	ADHD; premenstrual dysphoric disorder; posttraumatic stress disorder; stimulant use disorder (withdrawal)
Psychomotor retardation or agitation	Stimulant use disorder (withdrawal)
Hypomania or manic symptoms	Bipolar I disorder; bipolar II disorder
Hypersomnia or insomnia; sleep disturbance	Premenstrual dysphoric disorder; posttraumatic stress disorder; narcolepsy; stimulant use disorder (withdrawal)
Lethargy; fatigue; sleepiness	Premenstrual dysphoric disorder; circadian rhythm sleep-wake disorder; stimulant use disorder (withdrawal)
Lack of motivation	Cannabis use disorder
Change in appetite; change in food intake	Premenstrual dysphoric disorder; avoidant/restrictive food intake disorder; anorexia nervosa; bulimia nervosa; stimulant use disorder (withdrawal)
Weight loss	Anorexia nervosa
Cognitive deficits	Neurocognitive disorder
Psychotic features	Schizophrenia spectrum disorder
Catatonia symptoms	Schizophrenia spectrum disorder

ADHD, attention-deficit hyperactivity disorder; DSM-5, *Diagnostic and Statistical Manual of Mental Disorders, Fifth Edition*.

Supplemental Table 4. CANMAT Recommended Adjunctive Agents (Listed Alphabetically Within Each Line of Treatment).
Adapted with permission from Lam, et al. 2024.[4]

**Recommendation**	**Level of Evidence**	**Adjunctive Agent**
First line		
	1	Aripiprazole
	1	Brexpiprazole
Second line		
	1	Bupropion
	1	Intranasal esketamine
	1	IV racemic ketamine
	1	Olanzapine
	1	Quetiapine XR
	1	Risperidone
	1	Lithium
	2	Cariprazine
	2	Mirtazapine/mianserin
	2	Modafinil
	2	Triiodothyronine
Third line		
	3	Other antidepressant medications, including tricyclic class of agents

	3	Stimulants
	3	Lamotrigine
	3	Non-IV racemic ketamine
	3	Non-IV ketamine formulations
	3	Pramipexole
	3	Ziprasidone
Experimental		
	3	Psychedelic-assisted psychotherapy
Not recommended		
	n/a	Cannabis (insufficient evidence for efficacy; risk of harms)

Level of evidence:
1 = Meta-analyses with narrow confidence intervals and/or 2 or more randomized controlled trials (RCTs) with adequate sample size, preferably placebo controlled.
2 = Meta-analyses with wide confidence intervals and/or 1 or more RCTs with adequate sample size.
3 = RCTs with small sample size or nonrandomized, controlled prospective studies or case series or high-quality retrospective studies.
IV, intravenous; n/a, not applicable; RCT, randomized controlled trial; XR, extended release.

Supplemental Table 5. Goals and Principles for Managing Difficult-to-Treat Depression. Adapted with permission from McAllister-Williams et al. 2020[3]

**Treatment goals**	**Principles**
Achieve optimal symptom control using measurement-based treatment	Use of conventional treatments: first line medication, psychotherapy or neurostimulation
	– Antidepressant medication: increased dose, switch, or augmentation
	– High-intensity psychotherapy with/without medication
	– Alternatively: neurostimulation (eg, ECT)
	Use of nonconventional treatments: exercise, light therapy, novel medication and psychotherapies, neuromodulation
Target symptoms associated with poor outcome	Assess for and treat: anxiety, pain
Targeting symptoms to maximize function and quality of life	Assess for and treat: sleep problems, fatigue, cognitive problems
Manage comorbidities to reduce overall symptom burden → optimize long-term outcomes by ensuring adequate prophylaxis	Screen for and treat: physical health problems, substance misuse, comorbid mental illnesses, iatrogenic issues
Use self-management techniques to empower patients	Encourage skepticism of a pervasive negative view, behavioral activation, active community reconnection
	Encourage good sleep habits, exercise, good diet
	Enhance ability to cope with residual symptoms

	Occupational or interpersonal changes (adapt to capacities)
	Utilize online depression, anxiety and sleep management
Use integrated mental health services to help provide a sense of containment and ensure wide consideration of treatment options	Construct an individual management plan
	– Emphasize role of patient in long-term management
	– Establish a patient-centered pathway
Establish regular review of the patient's diagnosis and treatment	Formally assess severity of symptoms and impact on psychosocial functioning
	Reconsider diagnosis and screen for comorbidities
	Review predisposing, precipitating and perpetuating factors

ECT, electroconvulsive therapy.

Supplemental Figure 1. Early optimized treatment is critical for bringing people to full symptomatic and functional recovery.
Reprinted with permission from Habert et al, 2016.[5]

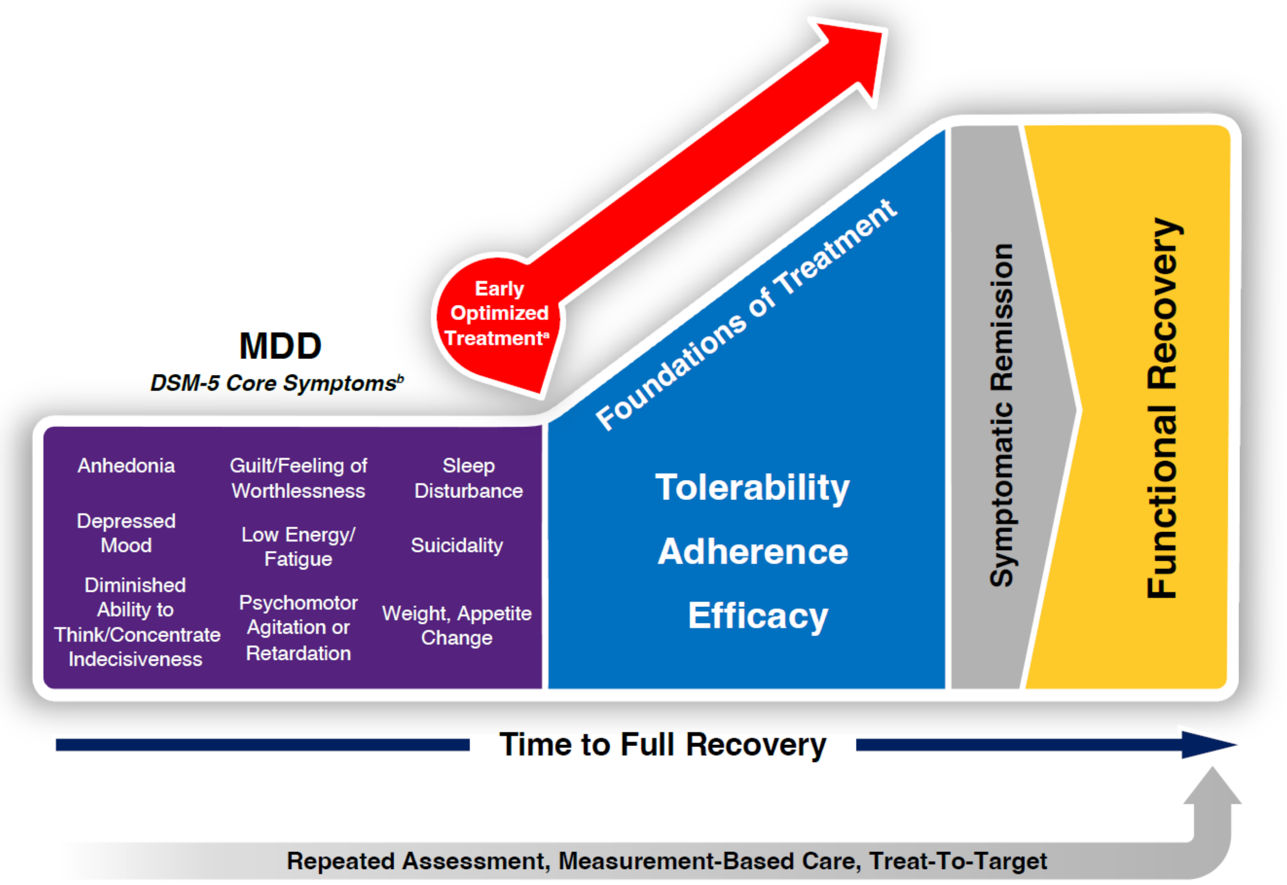

[a]Early diagnosis followed by rapid, optimal treatment.
[b]Alphabetical order.
DSM-5, *Diagnostic and Statistical Manual of Mental Disorders, Fifth Edition*; MDD, major depressive disorder.

References

1. Arnaud AM, Brister TS, Duckworth K, et al. Impact of major depressive disorder on comorbidities: a systematic literature review. *J Clin Psychiatry.* 2022;83(6).
2. Arnaud AM, Brister TS, Duckworth K, et al. Impact of treating depression on associated comorbidities: a systematic literature review. *Prim Care Companion CNS Disord.* 2023;25(1):22r03330.
3. McAllister-Williams RH, Arango C, Blier P, et al. The identification, assessment and management of difficult-to-treat depression: an international consensus statement. *J Affect Disord.* 2020;267:264-282.
4. Lam RW, Kennedy SH, Adams C, et al. Canadian Network for Mood and Anxiety Treatments (CANMAT) 2023 update on clinical guidelines for management of major depressive disorder in adults: Réseau canadien pour les traitements de l'humeur et de l'anxiété (CANMAT) 2023: Mise à jour des lignes directrices cliniques pour la prise en charge du trouble dépressif majeur chez les adultes. *Can J Psychiatry.* 2024;May 6 Online Ahead of Print:7067437241245384.
5. Habert J, Katzman MA, Oluboka OJ, et al. Functional recovery in major depressive disorder: focus on early optimized treatment. *Prim Care Companion CNS Disord.* 2016;18(5):e1-e11.
